# Algae-Derived C-Phycocyanin Mitigates AGE–RAGE-Induced ER Stress and Mitochondrial Apoptosis: Implications for Diabetes-Associated Neurodegeneration

**DOI:** 10.3390/ijms262211077

**Published:** 2025-11-16

**Authors:** Mei Chou Lai, Wayne Young Liu, Yu-Cheng Tzeng, I-Min Liu

**Affiliations:** 1 Department of Pharmacy and Master Program, Collage of Pharmacy and Health Care, Tajen University, Pingtung County 90741, Taiwan; 2 Department of Urology, Jen-Ai Hospital, Taichung City 412224, Taiwan; waynedoctor@gmail.com; 3 Collage of Nursing, Central Taiwan University of Science and Technology, Taichung City 412224, Taiwan; 4 Kaohsiung Medical University Hospital, Kaohsiung Medical University, Kaohsiung City 80708, Taiwan; hahahahanelson@gmail.com

**Keywords:** receptor for advanced glycation end products, advanced glycation end products, endoplasmic reticulum stress, C-phycocyanin, SH-SY5Y cells

## Abstract

Impaired glucose metabolism elevates the risk of neurodegenerative diseases by activating the receptor for advanced glycation end products (RAGE), thereby promoting oxidative and endoplasmic reticulum (ER) stress that leads to neuronal apoptosis. C-phycocyanin (C-PC), a natural pigment–protein complex derived from algae, possesses potent antioxidant and antiglycation properties; however, its capacity to modulate RAGE-mediated neurotoxicity remains to be fully elucidated. In this study, we established a RAGE-driven neuronal injury model by exposing differentiated SH-SY5Y cells to advanced glycation end products (AGEs; 300 μg/mL). Pretreatment with C-PC (15–50 μmol/L) improved cell viability, preserved neuronal morphology, and attenuated AGEs-induced oxidative stress, as indicated by reduced intracellular reactive oxygen species and mitochondrial superoxide levels. Furthermore, C-PC inhibited activation of the PERK-CHOP pathway, and upregulated Bcl-2 while downregulating Bax, thereby preventing cytochrome c release and reducing caspase-9/3 activation as well as apoptotic DNA fragmentation. These neuroprotective effects of C-PC were comparable to those observed with the selective RAGE antagonist FPS-ZM1. In conclusion, our findings demonstrate that C-PC confers robust protection against AGEs-induced neuronal injury by suppressing oxidative and ER stress pathways downstream of RAGE activation, highlighting its potential as a natural modulator of the AGE–RAGE axis for the prevention or treatment of diabetes-associated neurodegeneration.

## 1. Introduction

Individuals with impaired glucose metabolism, including those with impaired glucose tolerance or diabetes mellitus, are increasingly recognized to be at heightened risk for developing neurodegenerative diseases such as Alzheimer’s disease (AD), Parkinson’s disease, and vascular dementia [1,2]. These conditions share pathophysiological features including chronic oxidative stress, metabolic imbalance, and impaired neuronal resilience, which ultimately contribute to progressive cognitive decline, memory loss, and neurodegeneration [3,4]. A critical molecular mediator underlying this increased susceptibility is the receptor for advanced glycation end products (RAGE), which functions as a central hub integrating diverse neurotoxic signals [5]. In hyperglycemic states, the excessive formation and accumulation of advanced glycation end products (AGEs) activate RAGE, initiating a cascade of oxidative and inflammatory responses [6]. Notably, RAGE is not exclusively activated by AGEs; amyloid-β (Aβ), a key pathogenic hallmark of AD, also binds to RAGE with high affinity [7]. Engagement of AGEs or Aβ with RAGE facilitates its transport across the blood–brain barrier, promotes intracellular reactive oxygen species (ROS) generation, and augments neuroinflammatory signaling, thereby amplifying neuronal injury in a feed-forward loop [7].

Emerging evidence indicates that RAGE activation drives not only oxidative stress but also endoplasmic reticulum (ER) stress, characterized by the accumulation of unfolded proteins within the ER lumen [8]. Among the three canonical unfolded protein response pathways, the protein kinase RNA-like endoplasmic reticulum kinase (PERK)–eukaryotic initiation factor 2α (eIF2α)–activating transcription factor 4 (ATF4)–C/EBP homologous protein (CHOP) axis is a critical pro-apoptotic cascade [9,10]. PERK-mediated eIF2α phosphorylation suppresses global protein synthesis while selectively enhancing ATF4 translation, which subsequently induces CHOP expression [9,10]. CHOP disrupts the balance of B-cell lymphoma 2 (Bcl-2) family proteins, leading to mitochondrial dysfunction, cytochrome c release, and caspase-dependent apoptosis [11]. This cascade represents a critical mechanism by which ER stress precipitates mitochondrial apoptosis, ultimately driving neuronal loss [12]. Importantly, RAGE activation serves as a central upstream trigger of this process, tightly linking oxidative and ER stress to mitochondrial dysfunction and neuronal cell death [5]. Because RAGE responds to both AGEs and Aβ, targeting the AGE–RAGE axis has emerged as a promising strategy against diabetes-related cognitive decline and neurodegeneration [13,14]. FPS-ZM1 (4-chloro-N-cyclohexyl-N-(phenylmethyl)benzamide), a highly selective and high-affinity RAGE inhibitor, serves as the reference compound for RAGE blockade, owing to extensive experimental evidence demonstrating its robust neuroprotective effects and prevention of AGE- and Aβ-induced neurotoxicity without cytotoxicity in cellular and animal models [15,16]. However, the limited clinical translation of such inhibitors highlights the urgent need to identify novel, effective modulators of the AGE–RAGE pathway [17].

Algae-derived C-phycocyanin (C-PC) is a water-soluble pigment–protein complex belonging to the phycobiliprotein family, predominantly extracted from cyanobacteria such as Spirulina platensis and Plectonema species [18,19]. Structurally, C-PC consists of α- and β-subunits, each covalently bound to open-chain tetrapyrrole chromophores that confer its distinctive deep blue color and intrinsic fluorescence [20]. These properties have led to its widespread use as a natural colorant in food products including ice cream, beverages, chewing gum, and confectionery as well as in cosmetics such as lipsticks and eyeliners [21]. Beyond its applications as a coloring agent and immunofluorescent probe, C-PC has garnered increasing attention for its diverse biological activities, including antioxidant, anti-inflammatory, anti-apoptotic, hepatoprotective, and anticancer effects [22]. Of particular relevance is its antidiabetic potential: C-PC has been shown to stimulate insulin secretion, enhance glucose uptake, and suppress hepatic gluconeogenesis in various animal models and in vitro systems [23]. Notably, C-PC attenuates β-cell apoptosis induced by methylglyoxal and human islet amyloid polypeptide, while preserving insulin secretory function, suggesting its protective role against glucotoxic and lipotoxic insults [24,25]. In addition, C-PC has been reported to inhibit AGEs formation and lower serum levels of glycation markers such as Nε-carboxymethyllysine [26]. This antiglycation effect, coupled with its potent antioxidant capacity, raises the possibility that C-PC may interfere with the AGE–RAGE signaling axis; however, whether C-PC can directly inhibit RAGE activation or modulate downstream RAGE-mediated pathological processes remains unclear and warrants further investigation.

Given the pivotal role of RAGE in mediating hyperglycemia-induced oxidative and ER stress, investigating whether C-PC can suppress RAGE activation or modulate its downstream signaling pathways is of considerable translational relevance. Elucidating these mechanisms may support the development of C-PC-based therapeutic strategies to counteract RAGE-driven pathologies. Accordingly, the present study was designed to examine the regulatory effects of C-PC on RAGE activation and its associated stress-related signaling cascades under diabetic-like conditions. To establish a physiologically relevant in vitro model of AGE–RAGE-mediated neurotoxicity, SH-SY5Y neuroblastoma cells were employed [27]. These cells endogenously express RAGE and several neurodegeneration-associated proteins, and are highly responsive to stimulation by AGEs [28]. In this model, AGEs were applied to induce cellular stress and mimic the neurotoxic environment characteristic of diabetes [29]. The selective RAGE antagonist FPS-ZM1 was included as a pharmacological reference to benchmark the efficacy of C-PC. By clarifying the impact of phycocyanin on AGE-triggered RAGE signaling, this study aims to identify a potential therapeutic avenue for preventing or mitigating diabetes-associated neuronal injury.

## 2. Results

### 2.1. Phycocyanin Attenuates AGEs-Induced Cytotoxicity

**Figure 1:**
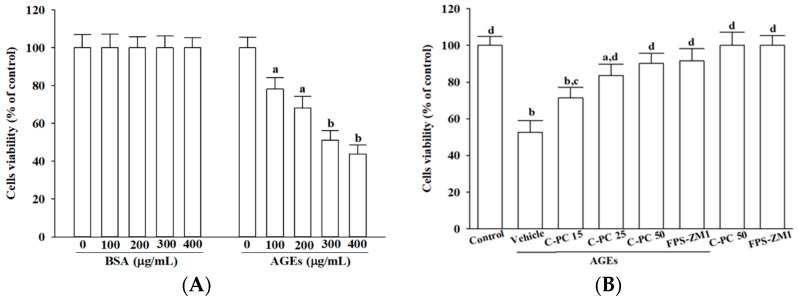
Effects of treatments on cell viability in AGEs-challenged SH-SY5Y cells. (**A**) SH-SY5Y cells were exposed to increasing concentrations of BSA or AGEs-BSA (AGEs) for 24 h to assess concentration-dependent cytotoxicity. (**B**) Cells were pretreated with C-phycocyanin (C-PC) at 15 (C-PC 15), 25 (C-PC 25), or 50 (C-PC 50) μmol/L, or FPS-ZM1 (100 nmol/L) for 1 h prior to incubation with or without 300 μg/mL AGEs for an additional 24 h. Cell viability was measured using the MTT metabolic assay and expressed as a percentage relative to untreated control cells. Data represent the mean ± SD from five independent experiments (*n* = 5), each performed in triplicate. ^a.^*p* < 0.05, ^b.^*p* < 0.01 vs. untreated control group; ^c.^*p* < 0.05, ^d.^*p* < 0.01 vs. vehicle-treated cells stimulated with AGEs.

SH-SY5Y cells were incubated with either BSA or AGEs for 24 h. Treatment with BSA across the tested concentrations (100–400 μg/mL) did not appreciably affect cell viability, whereas AGEs produced a concentration-dependent decline in cell survival. exposure to 300 μg/mL AGEs decreased viability by approximately 50%, and this concentration was therefore selected as the standard for subsequent cellular injury modeling (Figure 1A).

To evaluate cytoprotection, cells were pretreated with varying concentrations (15–50 μmol/L) of C-PC prior to 300 μg/mL AGEs exposure. C-PC demonstrated a clear concentration-dependent cytoprotective effect, with 50 μmol/L restoring AGEs-challenged SH-SY5Y cell viability to 90.2% of controls, an efficacy comparable to FPS-ZM1 (100 nmol/L), which increased viability to 91.6% (Figure 1B). Neither C-PC nor FPS-ZM1 affected the baseline viability of untreated cells (Figure 1B).

### 2.2. C-Phycocyanin Attenuates AGEs-Induced Neuronal Morphological Damage

**Figure 2:**
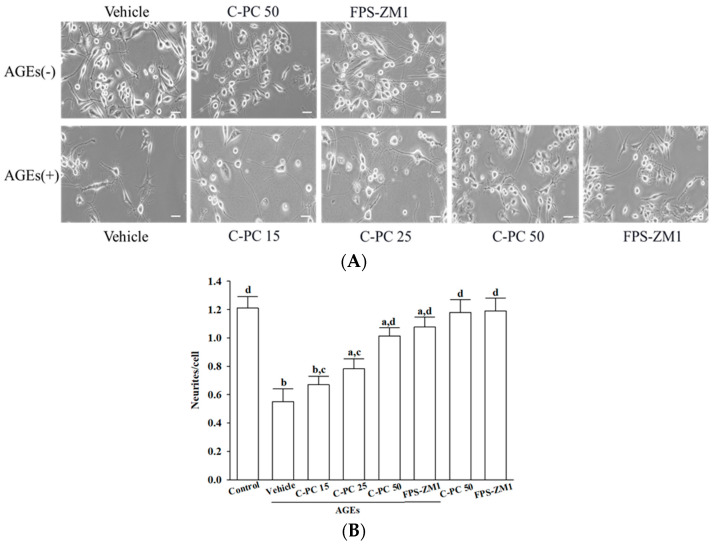
Effects of treatments on AGEs-induced morphological deterioration in SH-SY5Y cells. Cells were pretreated with C-phycocyanin (C-PC) at 15 (C-PC 15), 25 (C-PC 25), or 50 (C-PC 50) μmol/L, or FPS-ZM1 (100 nmol/L) for 1 h prior to incubation with (+) or without (−) 300 μg/mL AGEs for an additional 24 h. (**A**) Cell morphology was captured using an inverted microscope (Zeiss Axiovert 135). (**B**) Neurite outgrowth was quantified by counting the number of neurites extending from each soma, using ImageJ software (version 1.6.0). Data represent the mean ± SD from five independent experiments (*n* = 5), each performed in triplicate. ^a^ *p* < 0.05, ^b^ *p* < 0.01 vs. untreated control group; ^c^ *p* < 0.05, ^d^ *p* < 0.01 vs. vehicle-treated cells stimulated with AGEs.

In the absence of AGEs (300 μg/mL), treatment with C-PC (50 μmol/L) or FPS-ZM1 (100 nmol/L) did not promote neurite outgrowth or induce significant morphological alterations in SH-SY5Y cells, as neurite length, branching, and overall cellular morphology remained comparable to vehicle-treated controls under basal conditions (Figure 2A, upper row).

Exposure of SH-SY5Y cells to AGEs (300 μg/mL) led to marked morphological deterioration, as evidenced by extensive neurite retraction, cell rounding, and reduced cellular density (Figure 2A, lower row). Cells pretreated with increasing concentrations of C-PC (15, 25, and 50 μmol/L) progressively exhibited greater preservation of neurite length and network complexity, along with reduced cellular shrinkage compared to the AGEs group (Figure 2A, lower row). C-PC at 50 μmol/L afforded robust protection under AGEs-induced stress conditions, resulting in neuronal morphology that closely resembled that observed with FPS-ZM1 (100 nmol/L) treatment, as evidenced by elongated neurites and preservation of intact cellular architecture (Figure 2A, lower row).

Quantitative assessment of neurite outgrowth revealed that 300 μg/mL AGEs markedly suppressed neurite extension to 45.5% of control levels, whereas C-PC pretreatment enhanced neurite outgrowth in a concentration-dependent manner, with 50 μmol/L C-PC restoring outgrowth to 89.3% of control, an effect comparable to FPS-ZM1 (100 nmol/L), which elevated neurite outgrowth to 90.1% of control (Figure 2B).

### 2.3. C-Phycocyanin Reduces AGEs-Induced Oxidative Stress and Mitochondrial Superoxide

**Figure 3:**
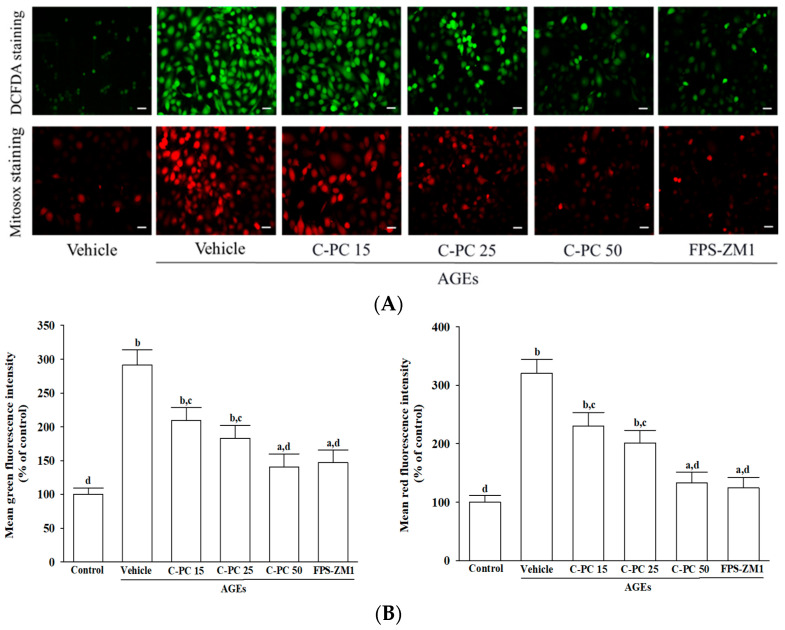
Effects of treatments on AGEs-induced oxidative stress in SH-SY5Y cells. Cells were pretreated with C-phycocyanin (C-PC) at 15 (C-PC 15), 25 (C-PC 25), or 50 (C-PC 50) μmol/L, or FPS-ZM1 (100 nmol/L) for 1 h prior to incubation with 300 μg/mL AGEs for an additional 24 h. (**A**) Representative fluorescence images illustrate intracellular ROS (upper panel), detected by DCFH-DA staining, and mitochondrial superoxide (lower panel), assessed by MitoSOX Red staining. Original magnification: 100×; scale bar: 100 μm. (**B**) Fluorescence intensity measurements were adjusted relative to total protein concentration, and results were expressed as a percentage of the untreated control group, which was defined as 100%. Data represent the mean ± SD from five independent experiments (*n* = 5), each performed in triplicate. ^a^ *p* < 0.05, ^b^ *p* < 0.01 vs. untreated control group; ^c^ *p* < 0.05, ^d^ *p* < 0.01 vs. vehicle-treated cells stimulated with AGEs.

Intracellular ROS levels were assessed using DCFH-DA fluorescence, and mitochondrial superoxide generation was vis evaluated by MitoSOX staining (Figure 3A). Baseline measurements in untreated SH-SY5Y cells revealed only faint green and red fluorescence, indicative of low basal oxidative stress. In contrast, exposure to AGEs (300 μg/mL) markedly increased both green and red fluorescence intensities, reflecting substantial intracellular ROS accumulation and excessive mitochondrial superoxide production (Figure 3A).

Fluorescence quantification (Figure 3B) confirmed that pretreatment with C-PC effectively attenuated AGEs-induced oxidative stress in a concentration-dependent manner. At 15 μmol/L, C-PC partially suppressed ROS and mitochondrial superoxide production, with further attenuation observed at 25 μmol/L, as indicated by progressively diminished fluorescence intensity. Notably, 50 μmol/L C-PC robustly inhibited AGEs-induced oxidative stress, resulting in 51.8% and 58.5% reductions in intracellular ROS and mitochondrial superoxide levels, respectively (*p* < 0.01 vs. AGEs; Figure 3B). Similarly, FPS-ZM1 (100 nmol/L) significantly suppressed AGEs-evoked oxidative responses, decreasing intracellular ROS and mitochondrial superoxide production by 40.5% and 61.2%, respectively (*p* < 0.01 vs. AGEs; Figure 3B).

### 2.4. C-Phycocyanin Protects Against AGEs-Induced PERK-ATF4-CHOP Apoptotic Cascade

**Figure 4:**
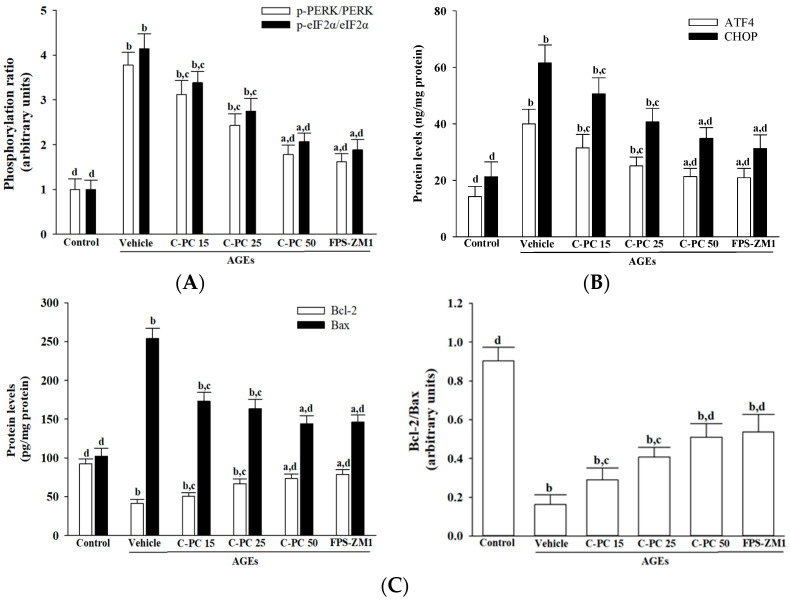
Effects of treatments on AGEs-induced activation of the PERK-ATF4-CHOP apoptotic cascades in SH-SY5Y cells. SH-SY5Y cells were preincubated for 1 h with C-phycocyanin (C-PC) at 15 μmol/L (C-PC 15), 25 μmol/L (C-PC 25), or 50 μmol/L (C-PC 50), or with FPS-ZM1 (100 nmol/L), followed by exposure to AGEs (300 μg/mL) for 24 h. (**A**) Immunodetection of phosphorylated PERK (p-PERK) and phosphorylated eIF2α (p-eIF2α), together with the calculated ratios of p-PERK/PERK and p-eIF2α/eIF2α. (**B**) Quantification of ER stress markers ATF4 and CHOP using ELISA assays. (**C**) Measurement of Bcl-2 and Bax protein levels, with calculation of the Bcl-2/Bax ratio to assess the balance between anti- and pro-apoptotic signals. Data are presented as the mean ± SD from five independent experiments (*n* = 5), each performed in triplicate. ^a^ *p* < 0.05, ^b^ *p* < 0.01 vs. untreated control group; ^c^ *p* < 0.05, ^d^ *p* < 0.01 vs. vehicle-treated cells stimulated with AGEs.

Exposure to AGEs (300 μg/mL) elicited pronounced ER stress in SH-SY5Y cells, as indicated by 3.8- and 4.1-fold increases in the ratios of phosphorylated PERK to total PERK and phosphorylated eIF2α to total eIF2α, respectively, compared to controls (*p* < 0.01; Figure 4A). Pretreatment with C-PC attenuated AGEs-induced activation of the PERK–eIF2α pathway in a concentration-dependent manner, as evidenced by substantial reductions in the p-PERK/PERK and p-eIF2α/eIF2α ratios, decreasing by 52.1% and 49.3%, respectively, at 50 μmol/L compared to AGEs-treated cells (*p* < 0.01; Figure 4A). Similarly, FPS-ZM1 (100 nmol/L) pretreatment effectively mitigated AGEs-induced ER stress, reducing these ratios by 42.8% and 45.4%, respectively (*p* < 0.01; Figure 4A).

Exposure to AGEs (300 µg/mL) markedly increased ER stress–associated proteins in SH-SY5Y cells, elevating ATF4 and CHOP expression to 2.8- and 2.9-fold, respectively, compared to controls (*p* < 0.01; Figure 4B). Pretreatment with C-PC effectively attenuated these increases in a concentration-dependent manner, with 50 µmol/L C-PC reducing ATF4 and CHOP levels by 46.6% and 43.4%, respectively, relative to the AGEs group (*p* < 0.01; Figure 4B). Similarly, the RAGE antagonist FPS-ZM1 (100 nmol/L) significantly mitigated AGEs-induced ER stress, lowering ATF4 and CHOP expression by 47.5% and 49.2%, respectively (*p* < 0.01; Figure 4B).

In SH-SY5Y cells, exposure to AGEs (300 µg/mL) markedly decreased Bcl-2 protein expression, an effect that was reversed by C-PC pretreatment in a concentration-dependent manner; specifically, 50 µmol/L C-PC elevated Bcl-2 levels to 1.8-fold that of the AGEs group (*p* < 0.01; Figure 4C, left panel). Similarly, FPS-ZM1 (100 nmol/L) pretreatment increased Bcl-2 expression to 1.9-fold relative to AGEs-treated cells (*p* < 0.01). C-PC also attenuated AGEs-induced Bax upregulation in a dose-dependent fashion, with 50 µmol/L C-PC reducing Bax expression by 43.1%, while FPS-ZM1 decreased Bax by 42.4% compared to the AGEs group (*p* < 0.01). Consequently, the Bcl-2/Bax ratio was restored by C-PC, with a 3.1-fold increase at 50 µmol/L relative to AGEs alone, and FPS-ZM1 achieving a similar 3.3-fold elevation (*p* < 0.01; Figure 4C, right panel).

### 2.5. C-Phycocyanin Inhibits AGEs-Induced Cytochrome c-Mediated Intrinsic Apoptosis

**Figure 5:**
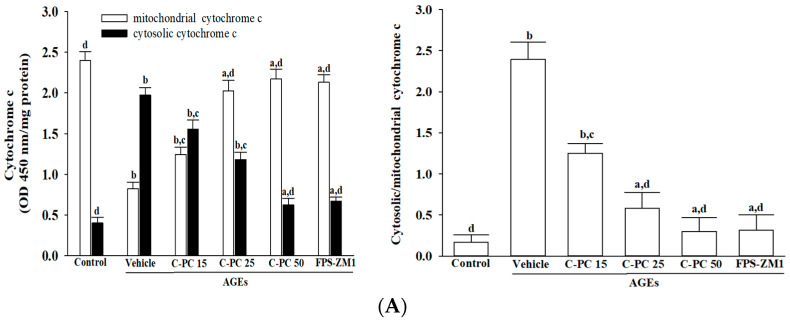

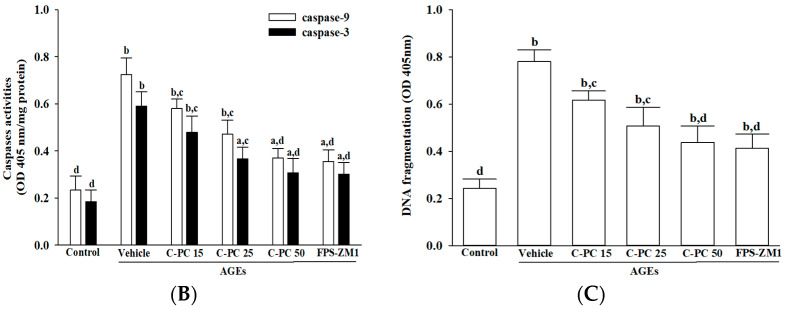
Effects of treatments on AGEs-induced cytochrome c-dependent caspase activation in SH-SY5Y cells. SH-SY5Y cells were pre-exposed to C-phycocyanin (C-PC) at concentrations of 15 μmol/L (C-PC 15), 25 μmol/L (C-PC 25), or 50 μmol/L (C-PC 50), or to FPS-ZM1 (100 nmol/L) for 1 h, followed by stimulation with AGEs (300 μg/mL) for 24 h. (**A**) Cytochrome c levels were analyzed in both mitochondrial and cytosolic fractions, and the cytosolic-to-mitochondrial cytochrome c ratio was calculated. (**B**) Caspase-9 and caspase-3 activities were assessed based on cleavage of their respective synthetic substrates, Ac-LEHD-pNA and Ac-DEVD-pNA. (**C**) Cytosolic histone-associated DNA fragmentation was quantified using a cell death detection ELISA. Data are expressed as the mean ± SD from five independent experiments (*n* = 5), each conducted in triplicate. ^a^ *p* < 0.05, ^b^ *p* < 0.01 vs. untreated control group; ^c^ *p* < 0.05, ^d^ *p* < 0.01 vs. vehicle-treated cells stimulated with AGEs.

AGEs exposure markedly induced cytochrome c release in SH-SY5Y cells, as evidenced by a pronounced reduction in mitochondrial cytochrome c accompanied by a robust increase in its cytosolic fraction (Figure 5A, left panel). Pretreatment with increasing concentrations of C-PC (15, 25, and 50 μmol/L) progressively restored mitochondrial cytochrome c levels while suppressing its cytosolic accumulation. At 50 μmol/L, C-PC afforded near-complete protection, comparable to FPS-ZM1 (100 nmol/L). Consistently, AGEs significantly elevated the cytosolic-to-mitochondrial cytochrome c ratio, confirming enhanced mitochondrial release, whereas C-PC pretreatment reduced this ratio in a concentration-dependent manner, with 50 μmol/L C-PC nearly normalizing values to control levels, mirroring the effect of FPS-ZM1 (Figure 5A, right panel).

Additionally, exposure to AGEs (300 µg/mL) strongly activated the intrinsic apoptotic pathway in SH-SY5Y cells, as indicated by significant increases in caspase-9 and caspase-3 activities to approximately 3.0- and 3.2-fold above control levels, respectively (Figure 5B). Pretreatment with C-PC attenuated this caspase activation in a concentration-dependent manner, with 50 μmol/L C-PC reducing caspase-9 and caspase-3 activities to 49.8% and 51.7% of the levels observed in the AGEs group. Comparable protective effects were observed with FPS-ZM1 (100 nmol/L), which decreased caspase-9 and caspase-3 activities to 49.1% and 50.2% of AGEs-induced values, respectively (Figure 5B).

Moreover, AGEs (300 µg/mL) exposure markedly increased apoptotic DNA fragmentation in SH-SY5Y cells, reaching approximately 3.2-fold above control levels (Figure 5C). This effect was significantly attenuated by C-PC in a concentration-dependent manner, with 50 μmol/L C-PC reducing DNA fragmentation by 44.1% relative to the AGEs group. Similarly, FPS-ZM1 (100 nmol/L) pretreatment decreased DNA fragmentation by 47.4% compared to AGEs-treated cells (Figure 5C).

## 3. Discussion

This study provides compelling evidence that C-PC, a natural biliprotein pigment derived from cyanobacteria, confers significant neuroprotection against AGE-induced neuronal injury through the coordinated suppression of RAGE-driven oxidative stress, ER stress, and intrinsic apoptotic signaling. Notably, our findings are the first to demonstrate that C-PC can attenuate PERK–eIF2α–ATF4–CHOP signaling and downstream mitochondrial apoptosis in a neuronal context, thereby establishing C-PC as a novel and mechanistically distinct natural modulator of the AGE–RAGE axis. This mechanistic insight significantly enriches the current understanding of C-PC-mediated biological activities, extending beyond its traditionally recognized antioxidant and antiglycation properties [21,22].

While previous research has predominantly focused on the metabolic benefits of C-PC—including amelioration of hyperglycemia, reduction in serum carboxymethyllysine, enhancement of antioxidant defenses, preservation of renal and retinal function in diabetic models, and protection of pancreatic β-cells from glucotoxic stress [23,24,25,26]—its effects on neuronal stress pathways have remained largely unexplored. In the present study, the neuroprotective actions of C-PC were primarily attributed to its suppression of oxidative and ER stress downstream of AGE–RAGE activation, rather than a direct inhibition of AGE formation. Although previous reports have demonstrated the antiglycation activity of C-PC in other biological systems, this property was not directly examined in our neuronal model. Our experimental design specifically aimed to clarify how C-PC modulates redox imbalance and ER-stress-related apoptotic signaling, which are key downstream consequences of RAGE activation. Accordingly, the present findings indicate that the protective efficacy of C-PC is largely mediated through attenuation of oxidative stress and stabilization of ER homeostasis. Future investigations incorporating quantitative measurements of AGE formation—such as ELISA-based or LC–MS-based analyses—will help determine whether antiglycation contributes directly to the observed neuroprotection. Such complementary studies will further elucidate whether C-PC acts at two mechanistic levels: (i) preventing AGE accumulation and (ii) mitigating oxidative and ER stress responses triggered by the AGE–RAGE axis. Collectively, these insights expand the therapeutic landscape for C-PC by linking its redox-regulatory capacity to neuronal protection under glycation stress. They also underscore its translational significance as a naturally derived compound with potential utility in preventing neurodegenerative processes associated with diabetes and chronic AGE–RAGE activation.

**Figure 6:**
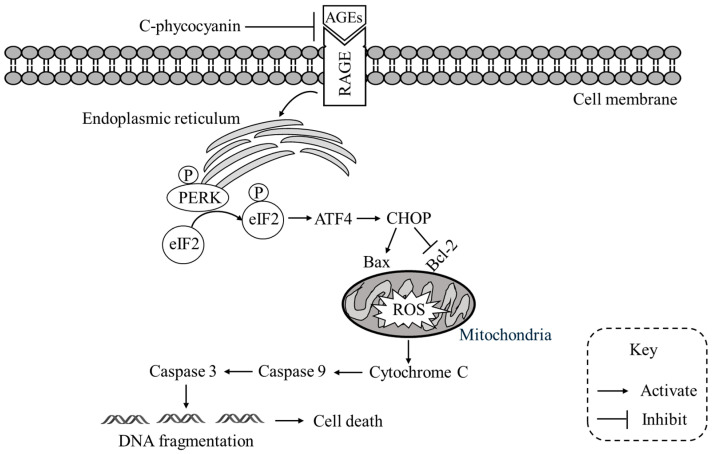
Proposed mechanism of C-phycocyanin against AGEs-induced apoptosis. AGEs binding to RAGE triggers ER stress and activates the PERK–eIF2α–ATF4–CHOP pathway, leading to Bax upregulation, Bcl-2 suppression, mitochondrial ROS accumulation, and cytochrome c release. This cascade activates caspase-9 and caspase-3, resulting in DNA fragmentation and apoptosis. C-phycocyanin blocks AGEs–RAGE interaction, alleviates ER stress, reduces ROS generation, and stabilizes mitochondrial integrity, thereby inhibiting caspase activation and preventing cell death.

Mechanistically, exposure to AGEs markedly elevated intracellular ROS and mitochondrial superoxide generation, reinforcing the established role of RAGE as a pivotal mediator of oxidative stress [4]. The resulting oxidative overload disrupted mitochondrial integrity, aggravated redox imbalance, and ultimately rendered neurons more vulnerable to injury [4]. These results are consistent with previous studies identifying the AGE–RAGE axis as a central driver of oxidative and mitochondrial dysfunction in neurodegenerative conditions [5,6,7], while providing further mechanistic insight into how glycation stress compromises neuronal survival. Importantly, pretreatment with C-PC effectively attenuated these deleterious effects in a concentration-dependent manner, underscoring its potent redox-regulatory capability. Beyond its antioxidant action, C-PC also mitigated ER stress by reducing PERK and eIF2α phosphorylation and subsequently downregulating ATF4 and CHOP expression. This coordinated suppression prevented the pro-apoptotic shift in Bcl-2 family proteins, preserved mitochondrial function, inhibited cytochrome c release, and blocked downstream caspase-9 and caspase-3 activation. Collectively, these results highlight the oxidative stress–attenuation mechanism by which C-PC preserves neuronal morphology, stabilizes mitochondrial integrity, and prevents RAGE-mediated apoptosis. The observed effects further suggest a potential hierarchical mechanism, wherein the previously reported antiglycation property of C-PC may act upstream to reduce AGE accumulation, while its antioxidant and ER stress-modulating activities exert direct cytoprotective effects once RAGE signaling is activated. The proposed mechanism through which C-PC counteracts AGE–RAGE-mediated ER stress and mitochondrial apoptosis is illustrated in Figure 6. Although the present study focused on the PERK–eIF2α–ATF4–CHOP pathway as the principal pro-apoptotic arm of the unfolded protein response, it remains plausible that C-PC may also influence other branches of ER stress signaling, including the inositol-requiring enzyme 1α and activating transcription factor 6 pathways. Future studies employing gene-silencing approaches or pathway-specific inhibitors will be essential to delineate these additional regulatory mechanisms. Moreover, the use of differentiated SH-SY5Y neuroblastoma cells confers translational relevance to this study, as these cells not only exhibit neuron-like morphology and functional characteristics but also endogenously express RAGE and display high susceptibility to AGE-induced stress [27,28]. Thus, this experimental model provides a physiologically relevant platform that closely recapitulates neuronal responses to glycation-induced injury, reinforcing the translational significance of our findings and supporting the potential of C-PC as a neuroprotective candidate targeting the AGE–RAGE axis.

To contextualize our findings, FPS-ZM1, a high-affinity and selective RAGE antagonist, was included as a pharmacological benchmark in this study [14,17]. Although FPS-ZM1 has consistently exhibited potent neuroprotective efficacy in diverse experimental models of AGE- and Aβ-induced injury, its clinical translation remains limited by concerns regarding long-term safety, metabolic stability, and restricted blood–brain barrier penetration [14,17]. In contrast, C-PC is a food-grade compound with well-documented safety and nutritional tolerability, representing a promising candidate for therapeutic repurposing [30]. Beyond its potential to interfere with RAGE signaling, C-PC exerts multifaceted biological activities, including potent antioxidant, antiglycation, and anti-inflammatory actions that converge on the mitigation of oxidative and ER stress as well as mitochondrial dysfunction [22,23,24,25,26]. These pleiotropic effects enable C-PC to provide broader and more physiologically integrated protection compared with the single-target blockade afforded by FPS-ZM1. However, the translational development of C-PC is constrained by its macromolecular size and hydrophilicity, which may limit systemic bioavailability and central nervous system penetration; thus, employing nanoformulation, peptide fragmentation, or intranasal delivery strategies may help overcome these pharmacokinetic barriers [31]. Collectively, the comparable efficacy of C-PC and FPS-ZM1 across multiple mechanistic endpoints underscores that C-PC functions as a natural, multitarget modulator of the AGE–RAGE axis, offering both mechanistic relevance and translational promise for the prevention or attenuation of diabetes-associated neurodegeneration.

Earlier studies have demonstrated that C-PC exerts protective effects in non-metabolic neurodegenerative conditions such as AD and ischemic brain injury primarily through antioxidant and anti-inflammatory mechanisms independent of receptor-mediated signaling [32]. In contrast, the present work elucidates a distinct and diabetes-specific mechanism in which C-PC directly modulates the AGE–RAGE axis to attenuate PERK–eIF2α–ATF4–CHOP-mediated ER stress and subsequent mitochondrial apoptosis. Nevertheless, certain limitations should be noted. First, although differentiated SH-SY5Y neuroblastoma cells provide a widely accepted neuronal model due to their endogenous RAGE expression and high susceptibility to AGE-induced injury, they cannot fully recapitulate the complex brain microenvironment, including interactions with glial cells, vascular networks, and systemic metabolic influences. Second, the present work primarily examined selected ER stress and apoptotic markers, while additional pathways such as autophagy, mitochondrial dynamics, and neuroinflammation may also play important roles in C-PC-mediated protection. Third, as this study was conducted in vitro, critical issues such as the pharmacokinetics, bioavailability, and blood–brain barrier permeability of C-PC remain unresolved, further in vivo investigations using models of diabetes and neurodegeneration will be essential to validate the therapeutic potential of C-PC. Moreover, while the current findings highlight the preventive efficacy of C-PC against AGE–RAGE-mediated neuronal injury, future investigations should also determine whether C-PC retains its neuroprotective activity when administered after AGE exposure. Such post-insult treatment paradigms would better simulate clinical scenarios in which neuronal injury has already been initiated by chronic hyperglycemia and glycation stress.

In conclusion, this study demonstrates that C-PC effectively attenuates RAGE-mediated oxidative stress and suppresses activation of the PERK-eIF2α-ATF4-CHOP axis of ER stress, thereby preserving neuronal morphology, maintaining mitochondrial integrity, and promoting cell survival under glycation-induced stress conditions. These findings provide a scientific basis for the further development of C-PC as a potential neuroprotective agent targeting AGE–RAGE–ER stress pathways in the context of diabetes-associated neurodegeneration and other RAGE-driven neurodegenerative disorders.

## 4. Materials and Methods

### 4.1. SH-SY5Y Cell Culture and Differentiation

Human SH-SY5Y neuroblastoma cells (CRL-2266; American Type Culture Collection, Manassas, VA, USA) were employed in this study. Cells were cultured in DMEM/F12 medium (Cat# SLM-243-B, Sigma-Aldrich, St. Louis, MO, USA) with 10% fetal bovine serum (FBS; Cat# F2379, Sigma-Aldrich, St. Louis, MO, USA), 1% nonessential amino acids (Cat#11140050, Thermo Fisher Scientific, Waltham, MA, USA) and the antibiotics, 100 units/mL penicillin and 100 µg/mL streptomycin (Cat#15140148, Thermo Fisher Scientific, Waltham, MA, USA) at 37 °C in a humidified incubator with 5% CO_2_. For neuronal differentiation, SH-SY5Y cells were seeded at 1 × 10^6^ cells/cm^2^ in 100 mm culture dishes containing DMEM/F12 with 10% FBS. When cultures reached 40–50% confluence, differentiation was induced by supplementing the medium with 10 μmol/L retinoic acid (Cat# R2625, Sigma-Aldrich, St. Louis, MO, USA). All experimental treatments were performed after a five-day differentiation period according to the previous study [33].

Neuronal differentiation and neurite outgrowth were documented using an inverted microscope (Zeiss Axiovert 135; Carl Zeiss, Oberkochen, Germany) equipped with an XY motorized stage and the “mark and find” module of AxioVision software (v4.8.1, Carl Zeiss GmbH, Thornwood, NY, USA). Neurite number was quantified by manually counting the processes emerging directly from the soma, and data were analyzed with ImageJ software (v1.6.0, NIH, Bethesda, MD, USA). Following differentiation, cells were reseeded at a density of 1 × 10^5^ per well in 6-well plates. Upon reaching confluence, cultures were dissociated using 0.05% (*w*/*v*) trypsin in phosphate-buffered saline (PBS; pH 7.4).

### 4.2. Treatment Protocols with AGEs and Pharmacological Modulators

Differentiated SH-SY5Y cells were plated in 6-well culture dishes at a density of 2 × 10^6^ cells per well to ensure sufficient material for subsequent biochemical analyses. Once the desired confluency was reached, cells were detached using 0.05% (*w*/*v*) trypsin in PBS (pH 7.4; Cat# P4474, Sigma-Aldrich, St. Louis, MO, USA). Stock solutions of bovine serum albumin (BSA; Cat# A8806, Sigma-Aldrich, St. Louis, MO, USA) and BSA-derived AGEs (AGEs; Cat# 121800-M, Sigma-Aldrich, St. Louis, MO, USA) were prepared in PBS at 1 mg/mL, filter-sterilized (0.22 μm), aliquoted, and stored at −20 °C. C-PC (Cat# 52468, Sigma-Aldrich, St. Louis, MO, USA) was dissolved in PBS to generate a 1 mmol/L stock, sterilized using a 0.2 μm syringe filter (Minisart^®^, Sartorius, Germany), and stored at 4 °C in the dark until use. FPS-ZM1 (Cat# 553030, Sigma-Aldrich, St. Louis, MO, USA) was dissolved in dimethyl sulfoxide (DMSO; Cat# 2650, Sigma-Aldrich, St. Louis, MO, USA) to yield a 10 mmol/L stock, aliquoted, and stored at −20 °C. All stock solutions were freshly diluted in culture medium before experiments, and the final DMSO concentration did not exceed 0.1% (*v*/*v*), a level previously reported to be non-cytotoxic [34]. Vehicle controls received medium containing an equivalent amount of DMSO.

For cell viability assessment, SH-SY5Y cells were treated with BSA or AGEs (100–400 μg/mL) for 24 h, either alone or in combination with C-PC (15, 25, or 50 μmol/L), concentrations previously reported to provide neuroprotection [32]. Based on viability results, 300 μg/mL AGEs reduced cell survival to ~50% of control levels and were selected for subsequent experiments. To evaluate cytoprotective effects, cells were preincubated for 1 h with C-PC (15–50 μmol/L) or FPS-ZM1 (100 nmol/L) [35], followed by exposure to 300 μg/mL AGEs for 24 h at 37 °C.

### 4.3. MTT-Based Determination of Cell Viability

Cell viability was evaluated using the 3-(4,5-dimethylthiazol-2-yl)-2,5-diphenyltetrazolium bromide (MTT) assay (Cat# M2003, Sigma-Aldrich, St. Louis, MO, USA) [36]. SH-SY5Y cells were plated in 96-well plates at a density of 5 × 10^3^ cells per well and cultured for 24 h to allow adherence. Cells were then treated for 24 h with BSA or AGEs (100–400 μg/mL), either alone or in combination with C-PC (5, 10, or 15 μmol/L) or FPS-ZM1 (100 nmol/L). After treatment, MTT solution (0.5 mg/mL in MEM) was added to each well and incubated at 37 °C for 4 h. The medium was removed, and the resulting formazan crystals were dissolved in 200 μL of DMSO. Absorbance was measured at 570 nm using a multimode plate reader (SpectraMax M5; Molecular Devices, Sunnyvale, CA, USA). Results were expressed as relative cell viability, with the untreated control group defined as 100%.

### 4.4. Measurement of Intracellular ROS

Intracellular ROS were assessed using the fluorescent probe 2′,7′-dichlorodihydrofluorescein diacetate (DCFH-DA; Cat# 35845, Sigma-Aldrich, St. Louis, MO, USA) [37]. After entering the cells, this non-fluorescent compound undergoes deacetylation followed by oxidation in the presence of peroxides, generating the fluorescent product dichlorofluorescein (DCF). Following the indicated treatments, cells were incubated with fresh medium containing 5 μg/mL DCFH-DA for 30 min at 37 °C. Fluorescence signals were visualized with a Leica fluorescence microscope (Leica Microsystems, Wetzlar, Germany). For quantitative evaluation, fluorescence intensity was measured using a microplate reader (SpectraMax M5; Molecular Devices, Sunnyvale, CA, USA) with excitation/emission at 488/525 nm, recorded every 30 min.

Following fluorescence measurement, the same wells were lysed, and total protein content was determined using the bicinchoninic acid (BCA) assay (Cat# ab102536; Abcam, Cambridge, UK). The fluorescence values (relative fluorescence units, RFU) were normalized to the total protein concentration (RFU/mg protein) to account for differences in cell density. ROS levels were expressed as relative fluorescence intensity, with untreated controls defined as 100%. Representative fluorescence images were captured from five randomly selected microscopic fields (100× magnification) in five independent experiments.

### 4.5. Measurement of Mitochondrial Superoxide

Mitochondrial superoxide production was determined using MitoSOX^TM^ Red, a fluorogenic dye selectively targeted to mitochondria (Cat# M36008, Invitrogen, Carlsbad, CA, USA) [38]. Once internalized, the probe is oxidized by mitochondrial superoxide to yield a bright red fluorescent product that enables selective detection of mitochondrial superoxide. Following the indicated treatments, cells were incubated with 5 μmol/L MitoSOX^TM^ Red for 10 min at 37 °C in the dark, followed by two washes with phosphate-buffered saline (PBS; pH 7.4) to remove unbound dye. Fluorescent signals were first visualized under a Leica fluorescence microscope (Leica Microsystems, Wetzlar, Germany) to verify mitochondrial localization and probe uptake. Quantitative measurements were then performed using a microplate reader (SpectraMax M5; Molecular Devices, Sunnyvale, CA, USA) at excitation/emission wavelengths of 396/610 nm, with readings recorded every 30 min.

After fluorescence quantification, cells within the same wells were subsequently lysed, and total protein levels were measured using the BCA assay (Cat# ab102536; Abcam, Cambridge, UK). The obtained fluorescence readings were adjusted according to protein content (RFU per mg of protein) to eliminate variations caused by minor differences in cell density. Mitochondrial superoxide generation was expressed as relative fluorescence intensity, with the untreated group set as 100%. Representative fluorescence micrographs were obtained from five randomly chosen areas at 100× magnification across five independent experiments.

### 4.6. Quantification of ER Stress Markers

Cells subjected to the indicated treatments were fixed in culture and analyzed for ER stress markers using commercial ELISA kits. PERK and phospho-PERK (Thr981) were measured with colorimetric detection kits (Cat# CB2027 and CB5548; Assay Biotechnology Inc., San Jose, CA, USA). eIF2α and its phosphorylated form (Ser51) were quantified using paired ELISA kits (Cat# CB5226 and CBP1538; Assay Biotechnology Inc., San Jose, CA, USA). ATF4 levels were determined with a kit from Signosis (Cat# TE-0039, Santa Clara, CA, USA), while CHOP was assessed with an ELISA kit from Fine Biotech (Cat# EM1933, Wuhan, China). Phosphorylation of PERK and eIF2α was expressed as the ratio of phosphorylated to total protein. ATF4 and CHOP concentrations were normalized to total protein and reported as ng/mg protein. Total protein content was determined by the BCA assay (Cat# ab102536; Abcam, Cambridge, UK).

### 4.7. Measurement of Mitochondrial-to-Cytosolic Cytochrome c Translocation

Mitochondrial outer membrane permeabilization was assessed by analyzing cytochrome c distribution between mitochondrial and cytosolic fractions. Following treatment, cells were harvested and homogenized in ice-cold isolation buffer. Subcellular fractions were obtained by sequential centrifugation: 800× *g* for 20 min to remove nuclei and debris, 10,000× *g* for 15 min to collect mitochondria, and 16,000× *g* for 25 min to obtain the cytosolic supernatant. Cytochrome c levels in each fraction were quantified using an ELISA kit (Cat# ab210575; Abcam, Cambridge, UK) according to the manufacturer’s protocol. Detection was based on antibody capture and horseradish peroxidase (HRP)-conjugated secondary antibody with chromogenic readout at 450 nm (SpectraMax M5; Molecular Devices, USA). Results were normalized to total protein content, determined by the BCA assay (Cat# ab102536; Abcam, Cambridge, UK).

### 4.8. Evaluation of Bcl-2 and Bax

The expression levels of Bcl-2 and Bax were quantified using specific ELISA kits (Bcl-2: Cat# CBCAB00158; Bax: Cat# CBCAB00157; Assay Genie, Dublin, Ireland) according to the manufacturer’s protocol. Briefly, cultured cells were fixed, permeabilized, and blocked with assay buffer for 1 h at room temperature, followed by incubation with primary antibodies for 2 h. After washing, horseradish peroxidase-conjugated secondary antibodies were added and incubated for 1 h. Colorimetric detection was performed using a chromogenic substrate for 10–20 min in the dark, and absorbance was measured at 450 nm with a microplate spectrophotometer (SpectraMax M5; Molecular Devices, Sunnyvale, CA, USA). Protein expression levels were normalized to total protein content, determined by the BCA assay (Cat# ab102536; Abcam, Cambridge, UK), and expressed as pg/mg protein. To evaluate the balance between pro- and anti-apoptotic signaling, the Bcl-2/Bax ratio was calculated by dividing the normalized Bcl-2 level by the corresponding Bax level in each sample.

### 4.9. Determination of Caspase Activities

The activities of caspase-9 and caspase-3 were evaluated using colorimetric assay kits (Caspase-9 Kit, Cat# APT139; Caspase-3 Kit, Cat# APT131; Sigma-Aldrich, St. Louis, MO, USA) based on the cleavage of their specific substrates acetyl-Leu-Glu-His-Asp-pnitroanilide (Ac-LEHD-pNA) and Acetyl-Asp-Glu-Val-Asp-p-nitroanilide (Ac-DEVD-pNA), respectively [39]. In brief, cell lysates containing 200 μg of protein were incubated with 200 μmol/L of the corresponding substrate in reaction buffer at 37 °C for 2 h. Cleavage of the substrate generated p-NA, which was quantified by measuring absorbance at 405 nm using a microplate spectrophotometer (SpectraMax M5; Molecular Devices, Sunnyvale, CA, USA). Caspase activity was normalized to total protein content determined by the BCA assay (Cat# ab102536; Abcam, Cambridge, UK) and expressed as OD_405_ per mg protein relative to vehicle-treated controls.

### 4.10. Evaluation of Apoptotic DNA Degradation

Apoptotic DNA degradation was quantified using a Cell Death Detection ELISA kit (Cat# 11544675001; Roche Molecular Biochemicals, Mannheim, Germany) [40]. Cytoplasmic fractions were prepared following the manufacturer’s protocol and applied to plates precoated with anti-histone monoclonal antibodies, enabling capture of nucleosome–histone complexes formed during apoptosis. Detection was carried out with a peroxidase-conjugated anti-DNA antibody, producing histone–DNA immune complexes proportional to DNA fragmentation. The colorimetric signal was developed with 2,2′-azino-bis(3-ethylbenzothiazoline-6-sulfonic acid) substrate for 10 min at 20 °C in the dark, and absorbance was measured at 405 nm using a microplate reader (SpectraMax M5; Molecular Devices, Sunnyvale, CA, USA). Values were normalized to total protein content determined by the BCA assay (Cat# ab102536; Abcam, UK) and expressed as OD_405_ per mg protein relative to vehicle-treated control samples.

### 4.11. Statistical Analysis

All experimental data are presented as mean ± standard deviation (SD), calculated from five independent biological replicates (*n* = 5), with each treatment group measured in triplicate wells. Statistical significance was assessed using one-way ANOVA. Where significant overall differences were detected, Tukey’s post hoc test was performed for pairwise comparisons. All analyses were carried out with SigmaPlot software (version 14; Systat Software, Inc., San Jose, CA, USA). A *p*-value below 0.05 was considered indicative of statistical significance.

## Data Availability

The original contributions presented in this study are included in the article. Further inquiries can be directed to the corresponding authors.
